# Releasing time to deliver care: a mixed methods evaluation of the implementation of enhanced midwifery continuity of carer

**DOI:** 10.1136/bmjopen-2024-095509

**Published:** 2025-07-28

**Authors:** Stephanie Gillibrand, Kate Parkyn, Charlotte Hall, Maartje Kletter, Elaine Harkness, Luke Aaron Munford, Paul Wilson, Jo Dumville

**Affiliations:** 1 The University of Manchester, Manchester, UK; 2 NHS England, London, UK; 3 NHS England and NHS Improvement South East, Reading, UK

**Keywords:** Health Equity, Midwifery, Health Services Accessibility

## Abstract

**Abstract:**

**Objectives:**

The enhanced midwifery continuity of carer (eMCoC) pilot programme provided additional resource (funding) to midwifery teams operating in the 10% most deprived areas in England. The eMCoC programme aims to provide additional support to those at greatest risk of poor maternal health outcomes. We conducted a rapid formative evaluation aiming to explore the implementation of the pilot programme to (1) generate timely insights to inform ongoing service delivery; (2) generate a logical framework of the eMCoC service and; (3) inform the design of a longer-term summative evaluation.

**Design:**

Rapid evaluation using mixed-methods.

**Setting:**

We explored implementation of the eMCoC service in 58 funded local midwifery teams across 23 Local Maternity and Neonatal Systems (LMNS). We undertook qualitative data collection in 10 case study sites across England, focusing on the implementation in 17 teams.

**Participants:**

We purposively sampled 34 service users who received care from enhanced teams, and 38 staff working in enhanced teams. Inclusion criteria for the service user interviews included women who had received care from enhanced teams during our evaluation period and were more than 28 weeks pregnant. Exclusion criteria included women who had not received care from our target teams. We undertook descriptive analysis using the Maternity Services Dataset to compare the characteristics of service users in enhanced teams with service users receiving other midwifery service models.

**Results:**

Many of the 58 teams funded were unable to implement eMCoC during the evaluation period because of institutional and organisational barriers. The barriers identified here are indicative of the barriers associated with implementing midwifery continuity of carer. Largely, the eMCoC service successfully targeted women living in the most deprived areas and a focus on reaching women living in these areas was valued by enhanced teams. Equally, enhanced teams strived to broaden the targeted characteristics (i.e. more broadly than on the basis of deprivation) to include a wider and more diverse set of social risk factors and vulnerabilities, based on local needs and priorities. Service users reported being well supported by the enhanced teams, including receiving relational and well-being support and personalised one-to-one public health education, information and support. Service users emphasised that enhanced teams went ‘above and beyond in their care’.

**Conclusions:**

Funding for eMCoC has been well received by both staff and service users. The implementation of the enhanced roles was perceived to have supported delivery of team-based care, facilitating successful release of midwifery capacity and the delivery of additional public health activities. Supporting a team-focused ethos seems an important feature of eMCoC services. This was consistent across sites and from both staff and service user perspectives. There appears to be many routes (i.e. different service delivery types) to delivering enhanced care, and the multiplicity of service delivery types found in this evaluation suggests no tightly prescribed way of meeting eMCoC’s objectives. The flexibility of the initial funding specification guidance from NHS England has been a key driver of local ownership and permitted eMCoC services to be organically built ‘from the ground up’. Our conclusions point to the value of autonomy afforded to local areas to use eMCoC funding as they deem necessary to best suit the needs of their staff and specific service user groups. Attention should be placed on the barriers to implementation and sustainability issues which can be addressed, namely: delays in releasing funding from LMNS and Integrated Care Boards to providers, and protecting maternity support worker and midwifery time to their allocated teams.

**STRENGTHS AND LIMITATIONS OF THIS STUDY**

- A strength of the study is a nationally representative sample across England.
- The study recruited a large qualitative sample and a diverse group of service users.
- A limitation of the quantitative analysis is the ability to only produce descriptive statistics due to the lack of statistical power associated with small populations receiving the intervention.

## Introduction

 There are persistent and ongoing inequalities in maternal, perinatal^1 2^ and neonatal^3^ health outcomes for ethnic minority women and women from deprived areas in the UK. While UK neonatal mortality is, on average, decreasing, 2019 data show the risk of stillbirth and perinatal mortality remains disproportionately high for babies born to women living in the most deprived areas.^1^ The Government’s National Maternity Safety Strategy^4^ sets out ambitious targets to reduce these inequalities. Maternity features in National Health Service (NHS) England’s health inequalities action plan, and Core20PLUS5^5^— an NHS England approach to support the reduction of health inequalities at both national and system levels.

The NHS Long Term Plan^6^ committed to roll out midwifery continuity of carer as the default model of care, available to all pregnant women in England by March 2023. Midwifery continuity of carer is a service delivery model that aims to provide consistent, personalised and safe care to women and their families via care from the same midwife (supported by a small team of midwives) throughout pregnancy, birth and the post-partum period. Implementation was to be targeted towards ethnic minority women and those living in the most deprived areas, to improve outcomes and reduce inequalities, as per the CORE20PLUS5 programme.^5^ In March 2022, however, the Ockenden Review^6^ called for NHS Trusts to review and suspend, if necessary, the continuity of carer model due to safety concerns around minimum staffing requirements.

In 2022, NHS England introduced an enhanced midwifery continuity of carer (eMCoC) pilot programme. This provided additional funding to teams delivering midwifery continuity of carer, operating in the 10% most deprived areas in England. The eMCoC programme’s focus reflects priorities identified in the Equity and Equality Guidance^7^ for Local Maternity and Neonatal Systems (LMNS), which includes a focus on accelerating preventative programmes that engage those at greatest risk of poor health outcomes prior to and during pregnancy and childbirth. Within the guidance, eMCoC is identified as a key innovation to address disparities in maternal and neonatal mortality and morbidity outcomes for groups including black, Asian and Mixed ethnic groups and women living in the most deprived areas. However, there is no identified academic evidence base that clearly identifies the eMCoC programme and its mechanisms that may impact on these outcomes.

Following a pre-pilot in nine LMNS in 2021/2022, eMCoC funding was made available to 58 midwifery teams in 23 LMNS in 2022/2023. Funding of up to £46,102 per team was awarded, the equivalent of one full-time equivalent (FTE) band 4 Maternity Support Worker (MSW). In their funding specification, NHS England noted that funding should provide ‘holistic support’ used to ‘reduce midwives’ workload and release additional time for midwives to care for women’ ^8^. There was flexibility in the specification for enhanced teams to decide on the additional staff they funded. This could include ‘creative approaches’ such as working with voluntary, community and social enterprise (VCSE) organisations to provide joined-up care, or the provision of extra staff including:

- MSWs, for example, those who speak languages of local communities, or to provide breastfeeding support.
- Link workers (who connect people to community-based support).
- Administrative workers.^8^

Eligibility for eMCoC funding was stipulated as areas that contain one or more of the 10% most deprived neighbourhoods in England (based on the Index of Multiple Deprivation) (IMD).^8^ The IMD is a neighbourhood measure of relative deprivation for areas inhabited by approximately 1–3000 people. The index is made up of 39 indicators spanning income, employment, education, skills and training, health, crime, barriers to housing and services, and the living environment. Deprivation is a known factor associated with adverse perinatal health outcomes.^9^ Drivers of perinatal health inequalities associated with living in a more deprived area include higher body mass index, smoking at birth, premature delivery, lower birth weight, lower breastfeeding rates etc.^10^,^13^

The Rapid Service Evaluation Team at the University of Manchester was commissioned by the National Institute for Health and Care Research to design and conduct an evaluation of the eMCoC pilot. The evaluation aimed to explore how the eMCoC programme was implemented, including how local contextual issues impacted on service implementation. We explored service access, acceptability, experience and impact, from the perspective of service users and staff. A list of full research questions can be found in the study protocol in online supplemental file 1.

## Methods

### Study design

We conducted a mixed-methods evaluation focusing on a sample of selected case sites, at NHS provider (Trust) level. For the qualitative workstream, we conducted individual in-depth interviews across 10 case sites with staff working in eMCoC services as well as service users who had received maternity care from eMCoC teams. Quantitative data were taken from the Maternity Services Dataset (MSDS) (more information about the dataset is provided here: https://digital.nhs.uk/data-and-information/data-collections-and-data-sets/data-sets/maternity-services-data-set) which contains routinely collected patient-level data, focusing on six case sites (due to data availability). MSDS captures information about activity carried out by NHS Maternity Services relating to a mother and baby(s), from the point of the first booking appointment until mother and baby(s) are discharged from maternity services. It also contains demographic information relating to ethnicity and geo-coded information on levels of deprivation of the home address.

To inform our evaluation design, we met with stakeholders including the NHS England Maternity and Neonatal Programme team, the National Maternity Voices Partnership and other VCSE organisations including the Parent-Infant Foundation. We also met with regional midwifery leads to informally explore wider contextual factors surrounding implementation of the eMCoC service nationally and regionally. Furthermore, we met with LMNS leads (or equivalent) covering 44 midwifery teams, to get a clearer overview of how services have been developed, designed, implemented and targeted to specific population groups. These discussions provided insights into how support is organised and functions in each region, as well as generating ‘soft intelligence’ relating to teams who were delivering the enhanced service.

We additionally collated and analysed available eMCoC funding documents, collated and reviewed available LMNS equity and equality plans to explore local maternity inequalities strategies and objectives and their fidelity to the plans surrounding eMCoC.

**Figure 1:**
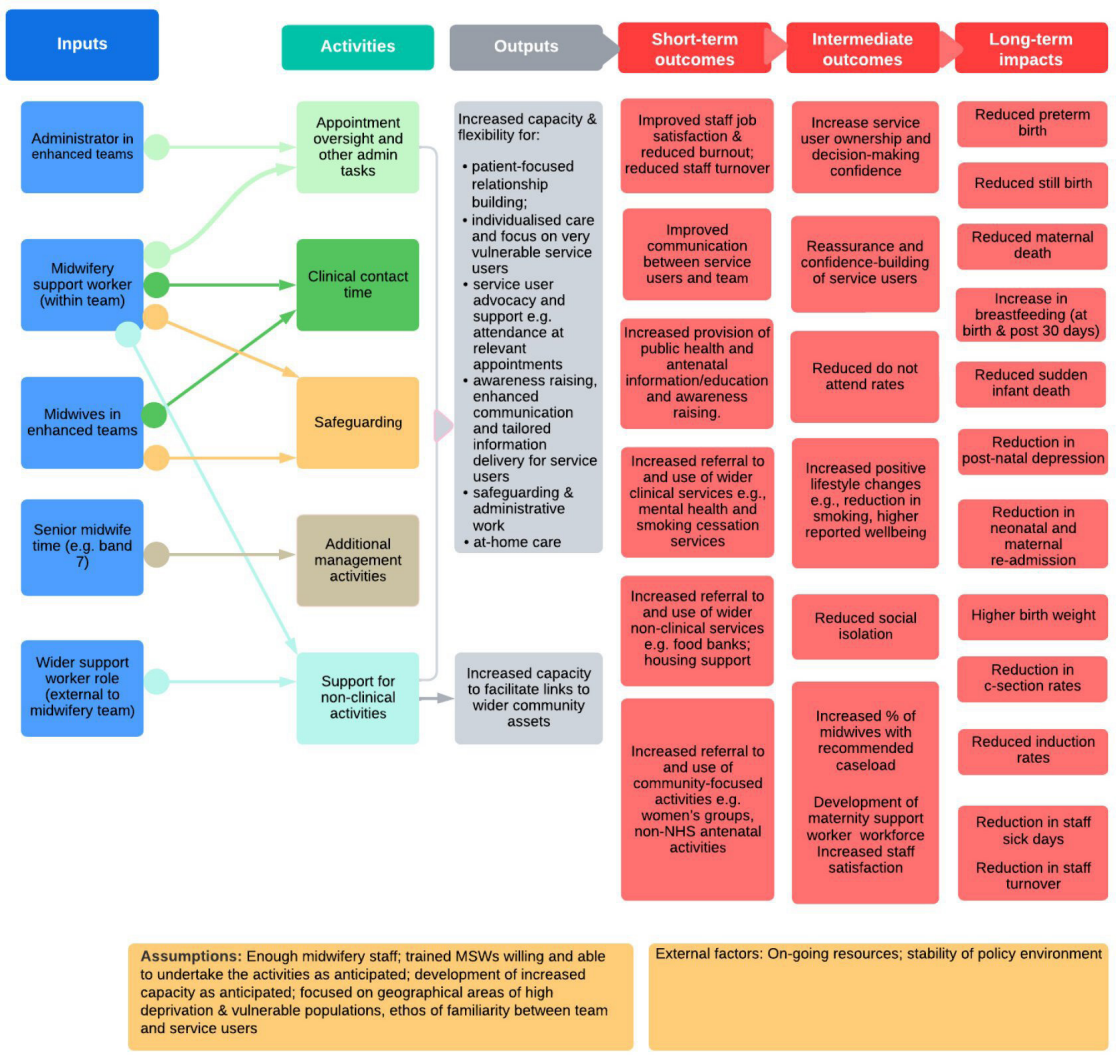
Logic model of the enhanced midwifery continuity of carer (eMCoC) service. The figure illustrates the logic process of change (in terms of outcomes and impacts) that may be anticipated from the midwifery continuity of carer (eMCoC) service. The six columns (left to right) display the logical process with key ingredients in each part of the logical process: inputs, activities, outputs, short, intermediate outcomes and long-term impacts. Each column is described in more detail below. Column 1 (left to right) ‘Inputs’ describes the key staff involved in an eMCoC service and can be described as the makeup of an eMCoC team. Not all eMCoC teams will have all of these staff members. For example, some teams may only have a Maternity Support Worker (MSW), some teams may only have an administrator and not an MSW. Column 2 ‘Activities’ describes the activities undertaken by eMCoC team members. The lines linking columns 1 and 2 depict which team members may undertake which activities. These activities link to the ‘Outputs’ described in column 3, where a crucial element of eMCoC is enabling greater capacity for the outputs listed in this column. Columns 3–6 display the anticipated outcomes and impacts from the inputs, activities and outputs detailed in columns 1–3. ‘Short-term outcomes’ are described in column 4, covering a range of workforce and service user level outcomes. These short-term outcomes may lead to the intermediate outcomes described in column 5. Finally, column 6 ‘long-term impacts’ describes some of the potential impacts which may occur over time.

From this sense-making work, we developed an initial logic model (see figure 1), which served as a tool to develop a shared understanding of the eMCoC programme and likely short-term, medium-term and long-term impacts on a range of outcomes. The logic model details the inputs, associated activities and expected outputs of eMCoC activities, and was iteratively shaped throughout the evaluation from this mapping work, the primary qualitative research, existing theory and discussion with wider stakeholders.

### Patient, public involvement and engagement statement

In the early stages of the evaluation, we set up a patient, public and VSCE study Advisory Panel to inform the evaluation activities and provide feedback and insights on preliminary findings. The group met five times during the course of the evaluation and provided inputs on the case study site selection, interpretation of emerging findings, and inputting on the design of the topic guides for the service user interviews.

### Selection of case study sites

10 case sites (with 17 midwifery teams) were selected to explore implementation of the eMCoC service and associated acceptability, experience and impact of the service on staff and service users. We employed a maximum variation sample to ensure variation in eMCoC service type, area-level characteristics, implementation status and service history. When we were recruiting case sites (October 2023), only 23 of the original 58 teams were ‘active’, reducing our sample pool for case site selection.

We included five sites who had no previous experience delivering eMCoC, sites that varied in starting the service (i.e. three sites who implemented the service later than other sites), one site which started and then discontinued the eMCoC service (due to challenges staffing the eMCoC position) and one site who had previously received pre-pilot funding but was not successful in the 22/23 funding round.

### Qualitative data

#### Data collection

From July 2023 to May 2024, we conducted 72 qualitative interviews with staff working in the eMCoC teams and service users receiving care by eMCoC teams. This sample size was justified on the basis of recruiting staff and service users from nine planned case study sites, aiming for four to five staff per site; however, because of difficulties in receiving local governance approvals and delays to service implementation, we increased the number of sites sampled to 10.

#### Service users

We purposively sampled women across six case study sites (Trusts) in teams where an eMCoC service had been implemented, aiming for a variation of antenatal and post-natal women. We were only able to recruit service users from six sites. This was due to (a) an inability to receive Trust-level approvals in one site due to a lack of capacity in the midwifery team and (b) the nature of recruiting from underserved, marginalised groups and known barriers to participating in research for these groups. The inclusion criteria were set at post-28 weeks duration of pregnancy to ensure a suitable amount of exposure to the eMCoC service. Interviews were informed by semi-structured topic guides informed by discussions with our Advisory Panel which focused on:

- Service users’ experience of care by the enhanced teams and how acceptable they found this, including positive and negative impacts/consequences of care.
- Service users’ priorities for their care during pregnancy and whether these were fulfilled.
- Whether service users’ felt like they had sufficient support, and comparisons with previous care (if applicable).

All interviews were conducted remotely (online via Microsoft Teams, telephone call or Zoom). The average duration of the interview was 19 min, with interviews ranging from 11 to 32 min. Four interviews were conducted in Farsi by a research staff member.

We asked participants to complete a demographic information sheet ahead of the interview. Descriptive characteristics of the sample are provided in table 1.

**Table 1:** Descriptive characteristics of enhanced midwifery continuity of carer (eMCoC) service users sampled in the qualitative data collection

	Total (n)
Gender	
Female	33
Missing	1
Age	
18–24	7
25–29	9
30–34	9
35–39	6
40–44	2
Missing	1
Disability	
Yes	3
No	30
Missing	1
Ethnicity	
Asian/Asian British	7
Black/black British	2
Mixed or multiple ethnic groups	2
White/white British	21
Other ethnic group	1
Missing	1
Index of Multiple Deprivation (overall domain) score	
Decile 1 (most deprived 10%)	8
Decile 2	5
Decile 3	4
Decile 4	4
Decile 5 or above (least deprived 50%)	11
Missing	2

##### Staff

We conducted 38 interviews with staff members across 10 case study sites. Interviewees included team midwives, team leads and continuity of carer lead midwives, MSWs, matron and head/deputy heads of midwifery, and other staff funded by eMCoC funding including administrators, Care Coordinators etc. The topic guides were informed by core constructs from the Consolidated Framework for Implementation Research applying Damschroder *et al*^14^ and the Health Disparities framework.^15^ Staff interviews explored staff views towards and acceptability of eMCoC, barriers and facilitators to implementing the service, the impact on staff and service users, anticipated outcomes and benefits on service users, and unintended consequences of the implementation of the eMCoC service. All interviews were conducted remotely (online via Microsoft Teams, telephone call or Zoom). The average duration of interviews was 31 min.

For both staff and service user interviews, we obtained informed (written or verbal) consent from participants and interviews were audio-recorded using an encrypted handheld device. Interviews were professionally transcribed by a university-approved supplier.

### Analysis

We rapidly analysed data from staff and service user interviews. Coding was conducted using a modified version of framework analysis.^16^ We identified themes and subthemes inductively, based on similarity,^16 17^ where themes were inductively deduced to generate a set of themes and subthemes. The themes and subthemes were subsequently added to a matrix (framework). An initial set of themes, based on the staff interviews, was discussed with the study’s Advisory Panel, the NHS England Maternity and Neonatal Programme team, and an Evaluation Sub-group monthly meeting, convened by NHS England, which included stakeholders such as regional and LMNS continuity of carer lead midwives, patient/public representatives and academic midwives. In these sessions, we focused on identifying potential gaps in the data and interpretation of the themes and subthemes, also considering key principles of the underlying theories (e.g. around inequity of service use/adoption, emerging themes around barriers/facilitators to implementation). These conversations led to an adapted thematic framework as we better understood the interpretation of themes and subthemes. Additionally, identified gaps were followed up in the next set of interviews conducted with service users and staff. A final set of themes from the staff and service user interviews was discussed again with the study’s Advisory Panel, with feedback provided around the interpretation of these and the reflections provided enabling greater context of the analysis.

### Quantitative data

We worked with NHS England data analysts who conducted descriptive analysis using the MSDS on service users in six sites (Trusts) where 14 enhanced teams implemented the enhanced service and had matched records in MSDS. We prioritised these six sites as we were able to confirm they had implemented the eMCoC service within our evaluation timeframe. We compared service user demographics (of all service users who had received care from enhanced teams) in our selected sites with service users accessing other midwifery service models used in other areas. The sample size for this data analysis was based on all service users (in the selected sites) who received care from eMCoC, midwifery continuity of carer, and neither eMCoC nor midwifery continuity of carer. The analysis provided aggregated descriptive statistics on eMCoC service users, midwifery continuity of carer service users and those receiving neither eMCoC nor midwifery continuity of carer (table 2) from 1 March 2023 to 29 Feb 2024.

**Table 2:** Definitions of service groups in the maternity services dataset

Teams delivering eMCoC	A pregnancy is recorded in MSDS as placed on a midwifery continuity of carer pathway by 29 weeks’ gestation, and the given Team Name matches an eMCoC Team Name provided by the research team, recorded at the relevant Trust.
Teams delivering midwifery continuity of carer	A pregnancy is recorded in MSDS as placed on a midwifery continuity of carer pathway (full MSDS data field criteria) by 29 weeks’ gestation, without a Team Name matching the Enhanced Team Names list provided by the research team.
All other (either midwifery continuity of carer or eMCoC)	A pregnancy is not recorded in MSDS as placed on a midwifery continuity of carer pathway (full MSDS data field criteria)—either through definitively declaring they were not placed on this pathway or due to incomplete data.

full MSDS data field criteria: midwifery continuity of carer data field criteria: having an antenatal care plan and a positive (‘Y’) midwifery continuity of carer pathway indicator recorded, along with a named Team and named Midwife recorded

eMCoC, enhanced midwifery continuity of carer; MSDS, Maternity Services Dataset.

The qualitative data collection and sense-making discussions also informed the priority set of selected variables for the descriptive analyses in MSDS. Insights from the qualitative data collection also provided important contextual information around the interpretation of findings from the quantitative analyses.

Data are presented by service group categories for a range of outcomes as detailed below using definitions taken from the Maternity Services Dataset User guidance^18^:

- Age at booking: age in years of the mother at the Booking Appointment.
- Ethnicity: based on the 2001 census values, the ethnicity of the mother in a maternity episode as specified by herself.
- Deprivation decile: decile of the Index of Multiple Deprivation 2019, derived using the Lower Super Output Area 2011 as based on the postcode of usual address as given by the mother.
- Complex social factors indicator: as identified at the Booking Appointment (binary Yes/No). Indicates if the mother is deemed to be subject to complex social factors, as defined by National Institute for Health and Care Excellance guidance (CG110 (https://www.nice.org.uk/guidance/cg110)).
- Support status indicator: as identified at the Booking Appointment (binary Yes/No). Indicates whether or not the mother feels she is supported during the pregnancy and with looking after a baby, from partner, family or friends.

Continuous variables (age at booking) are presented as mean and standard deviation, as well as median and inter-quartile range.

For categorical variables, the number and percentage within each category were provided by service model. Numbers are counts of unique pregnancies (so a woman with two pregnancies in the year would be counted twice, once per pregnancy). Percentages are calculated from the total count for that column category (eMCoC, midwifery continuity of carer or All other), not the overall total of pregnancies reaching 29 weeks’ gestation. For analyses, counts of zero were reported as zero, counts of 1–7 were rounded to 5 and all other counts were rounded to the nearest 5, with percentage rates (where relevant) calculated from rounded numbers. Therefore, totals may not add to 100%.

## Results

### Implementation of eMCoC

The evaluation was conducted between January 2023 and August 2024. At the time of recruiting case sites (October 2023), there were 23 (from the original 58 funded) teams who were active or planning to be active. As such, this context instigates a specific cohort of teams who:

- Were successful in receiving the funding, based on the selection criteria.
- Were successful in implementing the eMCoC service within the timescale of the evaluation.

Table 3 details our case study sites. Overall, we conducted qualitative interviews across 10 case study sites.

**Table 3:** Overview of case study sites

Region	No. of sites	No. of teams	eMCoC role
Midlands	1	2	MSW
Greater London	1	1	Administration support
North West	2	7	Care Coordinators; MSWs
North East and Yorkshire	1	1	Best Start in Life Advisors
	1	1	MSW
	2	2	MSW and administration support; MSW
South East	2	3	MSWs; administration support and midwifery time

eMCoC, enhanced midwifery continuity of carer; MSW, midwifery support worker.

Our case study sites also faced barriers to implementing the service. Many of the sites were slow to implement the eMCoC service, with three of our case study sites starting up to nine months later than anticipated. The barriers include wider organisational and organisational issues such as funding and staffing as well as the wider policy context. Delays in the transfer of eMCoC funding from Integrated Care Boards to provider level delayed implementation of eMCoC (specifically, the ability to advertise roles and hire new staff). Staffing was a wider barrier to eMCoC service implementation. Several sites struggled to maintain midwifery staffing levels, which impacted on starting planned service delivery or sustaining it once started. One site’s eMCoC service was halted as the enhanced staff member left and they were unable to hire a replacement because of hiring challenges and the short-term nature of the contract. Some staff in other sites also raised issues around MSWs being ‘borrowed’ by other community teams when they were short of midwives or MSW support. Broader policy challenges also played a significant role in the implementation of the eMCoC service. The Ockenden Review^7^ placed Trusts into disarray over the roll-out of midwifery continuity of carer, the delivery of which was the prescribed precursor to the eMCoC service, with several eMCoC teams paused or discontinued over the course of the evaluation.

#### Types of eMCoC services implemented

Table 4 presents the different eMCoC service delivery types and associated activities across the 17 teams in our 10 sites. Each service type describes how the additional eMCoC funding was used; we refer to the specific additional resource funded by the NHS England pilot as the ‘enhanced role’. Activities undertaken by these roles are what delineate these eMCoC services from midwifery continuity of care per se. Each service type’s characteristics are described to illustrate the contextual and nuanced variation between the service delivery types. The ‘History’ column details how the funded eMCoC roles relate to teams’ previous activities and thus provides important contextual information. It should be noted that no teams within our case sites demonstrated that they were using enhanced funds to fund colleagues in the VCSE sector or explicitly fund link-workers, although many of the tasks and activities undertaken by MSWs, Best Start in Life Advisors (BSiLAs) and Care Coordinators (described in more detail below) may include comparable aspects to a link worker role.

**Table 4:** Description of eMCoC services

eMCoC service	Enhanced roles	Summary characteristics of service	Description of activities delivered as part of enhanced role	History	No. of teams
**Service Type 1** MSW (role already existed in team)	MSWs only Directly service user facing	Service funded extra time of MSWs (increased their FTE or added another MSW)	Supporting antenatal and postnatal care (including postnatal visits). Support includes mental health and well-being, breastfeeding, signposting to non-clinical VCSE services, baby weight and checks, repeating bloods, chasing up referrals etc.	Teams have previously had MSW support prior to eMCoC funding, undertaking similar activities.	8
**Service Type 2** (MSW role—new to team)	MSWs only Directly service user facing	Service funded extra time of MSWs (increased their FTE or an additional MSW)	Supporting antenatal and postnatal care (including postnatal visits). Support includes mental health and well-being, breastfeeding, signposting to non-clinical (VCSE) services, baby weight and checks, repeating bloods, chasing up referrals etc.	Teams had **not** previously had MSW support in this way in these teams. The service is ‘new’.	2
**Service Type 3** (additional administration support)	Administration support only Indirectly service user facing	Service funds a separate administrator role	Booking women, re-arranging missed appointments, chasing up non-attenders, chasing up referrals etc.	Teams have **not** had a specific administrator role assigned/ supporting their team previously. The service is ‘new’.	1
**Service Type 4** (additional administration support combined with other role)	Administration support plus other service user facing role) Both directly and indirectly service user facing	Service funds a separate administrator role and another staff member (senior midwife (team lead) or MSW)	Booking women, re-arranging missed appointments, chasing up non-attenders, chasing up referrals etc. Administrator provides additional support for team leader or wider team through task-shifting.	Teams have **not** previously had an assigned administrator role. The service is ‘new’.	1
**Service Type 5** (other role)	Other support role (eg, non MSW or administrator roles Directly service user facing	Service funds ‘new’ roles, non MSW, midwives or admin: Care Coordinator or ‘Best Start in Life Advisors’ and these roles are separate to the specific team.	Supporting antenatal and postnatal care (including postnatal visits). Support includes mental health and well-being, breastfeeding, signposting to non-clinical (VCSE) services. Women are referred into enhanced support (automatically based on criteria or by midwives)	Some of these roles are new. These services were later to implement the service.	5

eMCoC, enhanced midwifery continuity of carer; FTE, full-time equivalent; MSW, midwifery support worker; VCSE, voluntary, community and social enterprise.

#### Facilitators of the enhanced service

In eMCoC Service Types 1 and 5, some teams described previous successful use of MSWs or other supporting roles in a similar capacity. Staff felt that the MSW role had usefully expanded and developed organically to provide increased and targeted support to the team and service users where it was needed most. In Service Type 1, it was reported that this development enabled close integration between the MSWs and midwives.

The success of the enhanced role (e.g. MSWs, administrators) was often put down to the personal characteristics of the staff members themselves, including a passion for the role, being willing to ‘go the extra mile’, initiative to develop the role, and independence.

### Description of service users accessing the eMCoC service

#### Descriptive insights from the MSDS analysis

Tables5^7^ present the descriptive analysis of the MSDS data. eMCoC service users (in our cohort of six case study sites) were more likely to live in the most deprived areas; 54% lived in the 20% most deprived areas (and 39% lived in the 10% most deprived areas). In comparison, 24% of service users receiving care under midwifery continuity of care (as recorded in MSDS) lived in the 20% most deprived areas (11% in the 10% most deprived areas). In terms of ethnicity, 62% of eMCoC service users in our cohort are White (52% White British), 15% of the cohort are Asian/Asian British and 11% are black/black British. In comparison, the service users recorded as receiving midwifery continuity of carer (no enhanced role) were recorded as 66% White, 17% Asian/Asian British and 7% Black/Black British. Indicators for complex social issues demonstrate that of eMCoC service users living in six case study sites, 16% had complex social issues, compared with 15% receiving care under midwifery continuity of carer (no enhanced role). The average age of mothers receiving eMCoC was also lower (29.1) compared with midwifery continuity of carer (30.5).

**Table 5:** Descriptive characteristics of women receiving different types of midwifery care, who reached 29 weeks’ gestation during March 2023 to February 2024: age and ethnicity

	Teams delivering eMCoC	Teams delivering midwifery continuity of carer	Either midwifery continuity of carer or eMCoC
Total	2815	99 590	500 250
Age			
Mean age (std. dev)	29.1 (6.1)	30.5 (5.7)	30.3 (5.6)
Median age (IQR)	29 (25–33)	31 (27–34)	30 (27–34)
Missing*	0 (0.0%)	5 (0.0%)	5 (0.0%)
Ethnicity			
White	1750 (62.2%)	65 320 (65.6%)	342 295 (68.4%)
Mixed	125 (4.4%)	2915 (2.9%)	13 750 (2.7%)
Asian or Asian British	430 (15.3%)	17 120 (17.2%)	79 030 (15.8%)
Black or black British	310 (11%)	7245 (7.3%)	32 870 (6.6%)
Other ethnic groups	150 (5.3%)	5085 (5.1%)	21 025 (4.2%)
Not stated†	55 (2%)	1760 (1.8%)	7745 (1.5%)
Missing	5 (0.2%)	145 (0.1%)	3530 (0.7%)

* Age at booking is based on two Mandatory data items so there are no missing entries. However, numbers not between 11 and 60 are considered outside reporting parameters and that is what is counted here.

† ‘Not stated’ category is a self-contained category, not part of the ‘Other Ethnic Groups’ category above.

eMCoC, enhanced midwifery continuity of carer.

**Table 6:** Descriptive characteristics of woman receiving different types of midwifery care, who reached 29 weeks’ gestation during March 2023 to February 2024: deprivation

	Teams delivering eMCoC	Teams delivering midwifery continuity of carer	Neither midwifery continuity of carer or eMCoC
Deprivation			
IMD decile 1 (most deprived)	1085 (38.5%)	10 960 (11%)	68 310 (13.7%)
IMD decile 2	435 (15.5%)	12 420 (12.5%)	60 075 (12%)
IMD decile 3	285 (10.1%)	13 625 (13.7%)	55 570 (11.1%)
IMD decile 4	295 (10.5%)	11 640 (11.7%)	52 450 (10.5%)
IMD decile 5	230 (8.2%)	9795 (9.8%)	49 425 (9.9%)
IMD decile 6	140 (5%)	9170 (9.2%)	48 185 (9.6%)
IMD decile 7	145 (5.2%)	8345 (8.4%)	44 080 (8.8%)
IMD decile 8	95 (3.4%)	8090 (8.1%)	43 075 (8.6%)
IMD decile 9	70 (2.5%)	8065 (8.1%)	40 070 (8%)
IMD decile 10 (least deprived)	30 (1.1%)	7305 (7.3%)	35 835 (7.2%)
Missing	10 (0.4%)	175 (0.2%)	3175 (0.6%)

eMCoC, enhanced midwifery continuity of carer; IMD, Index of Multiple Deprivation.

**Table 7:** Descriptive characteristics of women receiving different types of midwifery care, who reached 29 weeks’ gestation during March 2023 to February 2024: complex social and support status

	Teams delivering eMCoC	Teams delivering midwifery continuity of carer	Neither midwifery continuity of carer or eMCoC
Complex social factors indicator			
Yes	455 (16.2%)	14 735 (14.8%)	61 320 (12.3%)
No	2365 (84%)	84 800 (85.1%)	420 860 (84.1%)
Missing	0 (0%)	55 (0.1%)	18 065 (3.6%)
Support status indicator			
Yes	2710 (96.3%)	86 045 (86.4%)	379 675 (75.9%)
No	55 (2%)	1215 (1.2%)	10 750 (2.1%)
Missing	55 (2%)	12 330 (12.4%)	109 825 (22%)

eMCoC, enhanced midwifery continuity of carer.

#### Insights from the qualitative interviews

When conducting interviews with staff regarding the populations they served, we found that there was some pushback from sites on the eMCoC funding criterion that stipulated that teams should solely target care to service users based on their postcodes. It was felt that as a result of this, other social risk factors and vulnerabilities were de-prioritised as a result, and vulnerable communities and other at-risk groups could miss out on the support provided by the enhanced service. For instance, some sites indicated that targeting care on low-income postcodes did not necessarily reach the most vulnerable groups in highly urban areas like London, nor enabled them to reach ethnic minority women in other areas. Within sites, some teams served a range of mixed affluence postcodes that meant the enhanced team’s resource was at risk of being spread across a range of service users from these mixed affluent areas.

Alongside deprivation and ethnicity, staff in most sites also emphasised targeting service users with other types of social risk factors and additional vulnerabilities, based on identification of local need and understanding of vulnerabilities: people facing insecure housing and financial issues, asylum seekers and refugees, non-English language speakers, traveller communities, people with mental health conditions, non-attenders and people with complex social issues (including high rates of smoking, drug and alcohol dependency issues, and homelessness). Some sites emphasised the high rates of safeguarding and social service involvement of their caseload: three teams in three different sites described that up to 70–85% of the service users within their caseload had some degree of safeguarding concerns or responsibilities. Younger parents were also raised as a specific vulnerability, with one site (one team) targeting young parents only (under 21), within a large geographical area.

### Staff acceptability, experiences and impact of the enhanced service

The following sections describe staff views towards the service, reporting on the acceptability of the enhanced service through the value of more time through increased capacity and increased flexibility. Staff highlighted positive experiences of the enhanced roles and the impact of the enhanced service, enabling the targeting of care to service user needs.

#### Increasing midwives’ capacity through the use of additional roles

Across our case study sites, staff positively reported that capacity increased for midwives (as anticipated in the logic model, see ‘Outputs’, column 3, figure 1) because of the funded enhanced roles. This was largely because the enhanced roles were usefully undertaking tasks for the midwives. For example, MSWs assisted with antenatal clinics (blood pressure, urine tests, taking blood, glucose tests) and pre-booking clinics, as well as doing postnatal visits (day 3 and day 5 visits, weighing and re-weigh visits, breastfeeding support, jaundice checks, blood spot tests etc.). MSWs also supported midwives with administrative tasks (see ‘Activities’ in the logic model, column 2, figure 1). It was noted that eMCoC-funded administrator roles (Service Types 3, 4) increased midwives’ capacity by re-booking appointments, helping to manage the midwives’ diaries and following up with non-attenders and referrals.

In eMCoC Service Types 1, 2 and 5, enhanced roles (i.e. MSWs and BSiLAs) also took on some of the antenatal public health support and education. In two sites, MSWs ran antenatal breastfeeding support groups or organised frequent antenatal sessions, providing public health information and practical advice around safe sleeping, bathing and breastfeeding etc. in local community spaces (see ‘Outputs’ in the logic model, figure 1). Service users and staff also reported numerous examples of MSWs providing one-to-one breastfeeding support (on some occasions alongside group classes) and doing specific at-home repeat visits to provide breastfeeding support. In two sites, it was identified that without the MSW in post to provide these antenatal breastfeeding classes and ‘baby clubs’, they were unlikely to happen.

#### The value of ‘more time’ via increased capacity

As judged by staff, enhanced teams (i.e the enhanced roles and midwives) are perceived to deliver better team-based care by doing more because of the additional capacity provided by the enhanced role. There was a sense that this team-based ‘enhanced’ care, provided by both midwives and the enhanced role, went above and beyond what was being delivered before, where elements related to public health and parent education were able to be more thoroughly addressed (see ‘Inputs’ and ‘Activities’ in the logic model, figure 1, columns 1, 2).

There was a sense that additional support provided by eMCoC reassured midwives that important non-clinical aspects were covered more completely than they would have otherwise been. It was perceived that this supports better care, less pressure and reduced workload on the midwives (especially in terms of unpaid overtime), which may lead to reduced burnout (as depicted in ‘Outcomes’ in the logic model, figure 1, column 4). A key element as emphasised by staff was that the enhanced role created extra capacity, ensuring that aspects of patient care were less likely to get missed.

they are the glue in the team, aren’t they? They seal the cracks, they [ ] liaise with people and follow things up and chase things up. (INT_01)

[MSW] does the checks on the ladies and the babies when she goes there, so nothing goes unmissed really. (INT_03)

Staff were overwhelmingly positive about the enhanced roles, describing them as ‘part of the team’, leading to a heightened sense of team functionality and better working arrangements. Service Types 1–4 enhanced roles (i.e. MSWs and administrators) were described as ‘invaluable’ and ‘essential’; ‘a lifesaver’ to the team.

I know we’re quite lost without her, if I’m honest. I would hope that would be the same if it wasn’t her, I’m sure it would be. Because the role they do is incredible…she’s a lifesaver (INT_019)

we need them basically, they’re amazing. [ ] They are definitely invaluable (INT_021)

#### Increased flexibility and targeting of care to service user needs

Staff highlighted that the eMCoC service allowed both midwives and enhanced roles (ie, MSWs, Care Coordinators, BSiLAs) to provide flexible, tailored care and individualised support. This included flexibity for enhanced team members (i.e. both midwives and enhanced roles) to provide holistic support responsive to women’s needs: doing at-home appointments, providing one-to-one breastfeeding support, doing numerous and repeat postnatal visits for breastfeeding support, doing extra antenatal appointments, providing tailored information, and keeping postnatal women on their caseload longer. These are detailed in the anticipated outputs in the logic model (see ‘Outputs’, column 3, figure 1). It was highlighted that the MSW role was especially flexible, and this enabled the MSWs to provide tailored support including more time with women with specific needs.

Additional administrative support was viewed as giving midwives valuable time to meet the additional timely requirements of the increased needs and support demands of high-risk, target groups. For instance, the onerous administration related to safeguarding and involvement with children’s services, using interpreters in midwifery appointments, and additional administrative burdens created from disproportionately high rates of non-attenders in these groups. It was reported that vulnerable groups required greater one-to-one and relational support and the additional capacity enabled midwives with more time to better support vulnerable service users, for example, to facilitate follow-up on sensitive issues and concerns such as mental health or domestic abuse, undertake additional home visits and offer extra appointments.

### Service user perspectives and staff-reported service user experiences

Service users reported feeling well supported by enhanced teams. In terms of the eMCoC roles themselves, the BSiLAs were met positively, where well-being and relational support was especially highlighted. The MSWs in Service Type 1 who provided one-to-one, at-home and group breastfeeding and education classes were also highlighted as particularly valued by service users in terms of being supportive and informative. Some service users emphasised they had received a relational form of care from enhanced teams, were made to feel comfortable and received well-being support by the enhanced roles. Service users felt the team members were invested in their care, and that they had built a good relationship them. This was also echoed by the insights from staff. Service users also emphasised that their experience of receiving care from enhanced teams was more positive because of these relational factors, in comparison to the care received from other teams.

I went to two workshops post-, before pregnancy, when I was pregnant, I went to the breastfeeding one with [MSW name], and I went to a birth and labour one. And I feel like I came out with a lot of knowledge that I didn’t know and a lot of confidence as well with breastfeeding. There was a lot that I found out about breastfeeding that I didn’t know and if I didn’t know those things, I might have given up on breastfeeding a lot earlier. I’d heard lots of stories of ladies not having the knowledge. (SU_15)

I really struggled after having her, with how I felt. So she was a big, big support. And then on discharge [ ] actually, because I had a few concerns with her, the baby, [MSW] actually took me serious and got things in place to be tested and that. So she could see where I was coming from (SU_18)

Service users and staff described how service user facing enhanced roles provided signposting to other non-clinical services. In Service Types 1, 2, 4 and 5, those in the funded enhanced roles directly used links to other services (including the VCSE sector) to source baby equipment through baby basics charities and specialist charities (e.g. support for asylum seekers). They also signposted service users to other support services including breastfeeding networks, mother and baby groups, foodbanks, maternity grants and financial support charities, mental health and well-being support, domestic violence support, housing support services, bereavement support and charities, children’s centres etc. This is described in the outputs and short-term outcomes in our logic model (figure 1, columns 4–6).

It’s a very good job that [MSW name] is doing, just, you know, the wellbeing. She asked all the questions, whether you are okay, do you have enough support? So I just love the way that she asked me. I mean, I can feel the support from her. I have got a very bad back pain and I said to [MSW] that I’m having the pain. She immediately forwarded the email that she has got from physio and she asked me to go through them. If I have any questions, she answers immediately. (SU_10)

A small minority of service users reported some negative experiences from the care provided by enhanced teams, around a lack of clear communication, for example, issues with specific electronic records or linked digital resources, lack of follow-up and issues with contacting midwives.

## Discussion

We identified five different eMCoC service delivery types across 10 sites, driven by contextual difference and nuances in implementing sites, largely related to their historical setup. For instance, five teams across three of our case study sites described working as an enhanced team prior to the pilot—by focusing on high-need groups, and most teams in our case study sites had previously had a MSW role. Despite this variation, it is striking that the different enhanced roles and services undertake similar activities consistent across the various services including: signposting to additional services, public health education and support and supporting midwives with clinical and non-clinical duties. There appears to be many routes (i.e. different service delivery types) to delivering enhanced midwifery care, and the multiplicity of services found in this evaluation suggests no tightly prescribed way of meeting eMCoC’s objectives. This can be viewed as a direct, and positive, implication from the flexibility of NHS England’s specification and implementing guidance,^9^ which provided local sites the flexibility required to ensure delivery of the essential functions of eMCoC.

As expected, there was an over-representation of mothers who received eMCoC living in the most deprived areas. However, there was little evidence of differences in ethnicity composition across delivery modes, although there was a slightly higher proportion of Black mothers receiving eMCoC compared with MCoC. It is unclear whether this is driven by geography (ie, targeted on deprivation) or by an explicit focus on targeted ethnic minority groups. Insights from the qualitative data and MSDS support the descriptive data that enhanced teams placed an additional emphasis on reaching women with other types of social needs and risk factors.

The eMCoC service enabled increased capacity for midwives, which was widely positively supported by staff. It was felt that this enabled better care (at the team level), by doing more of what otherwise may not have been done as thoroughly (i.e. public health education, information and service user support) which reduced potential burnout in enhanced teams. The service also enabled all team members to be more flexible with their time to better support the teams of high-risk target groups. We found positive views towards the eMCoC service from both staff and service users. Service users reported being well supported by the enhanced teams, including receiving relational and well-being support and personalised one-to-one public health education, information and support. Service users emphasised that enhanced teams went ‘above and beyond in their care’.

This study is the first peer-reviewed study in the UK to explore an ‘enhanced’ service of midwifery continuity of carer that funds a specific additional staff member (or equivalent). In South London, similar initiatives have developed midwifery-focused targeted care to ethnic minority populations,^19^ and this initiative also focused on providing holistic care to infants and their families.^20^ While services which replicate the eMCoC service are not discussed explicitly in the literature, one Australian study^21^ discusses a midwifery continuity of carer model alongside ‘wrap-around’ services, which may be the closest manifestation of an ‘enhanced’ midwifery continuity of carer service identified in peer-reviewed international literature. In this 'wrap-around model, link workers and administrative capacity provide additional support around cultural relevance, cultural safety and cultural sensitivity. The rationale behind a focus on cultural needs is echoed in other studies in which translation support and cultural representation through a midwifery continuiy of carer model is reported to be important for under-served groups, in particular ethnic minority women.^22^ Our study is the first study globally (that we are aware) to explore the implementation of this service from a range of perspectives (i.e. from both staff and service users).

Currently, no logic model exists which theoretically demonstrates the processes and activities which may lead to better clinical outcomes for deprived population and ethnic minority groups, and those with social risk factors, receiving care under an eMCoC service. The literature demonstrates that midwifery continuity of carer models (also sometimes referred to as ‘caseload midwifery’) could have positive impacts on birth, neonatal and perinatal outcomes for under-served groups,^1921 23^,^26^ as well as improvements in health behaviours, including smoking cessation, among women.^26^ However, many of these studies are conducted in specific settings and contexts, that is, in Australia, with indigenous groups and have limitations including small sample sizes. There is limited evidence from the UK, with small comparative studies suggesting that ethnic minority and socially disadvantaged women and women with social risk factors who receive midwifery continuity of carer models of care may have better birth outcomes than those without continuity of care.^27 28^ The model was reported to have the greatest impact in the highest risk populations, that is, mothers in areas of more deprivation (measured by IMD) and those from ethnic minority backgrounds.^19^ Our logic model is the first of its kind looking at how an enhanced service’s processes and activities may lead to better clinical outcomes. This will be explored further in a longer-term evaluation.

### Strengths and weaknesses of the study

Strengths of this study include the large sample size of interview respondents. We also recruited a diverse group of service users, including undertaking four interviews in Farsi, and a wide range of staff in teams including midwives, heads of midwifery, maternity support workers, etc.

Limitations include the reduced pool size for our case study site selection. As of October 2023, when we were recruiting case sites, only 23 teams (of the initial 58 teams offered funding) were active or planning to be active. This reduced our sample pool size for case site selection significantly and means that sites may not be fully representative of the full cohort of funded sites. This presents a possible selection bias in that sites that did start may be those best placed to implement eMCoC services. Nevertheless, it is clear that sampled case study sites had challenges in implementing the service, and thus the findings presented here provide essential learning for the roll-out of other pilot programmes and the ongoing enhanced programme.

As we were limited to the sites we could recruit from (i.e from the limited pool of those that had implemented the service), a further limitation was the potential for selection bias by clinical team members on whom we had to rely on to identify potential eligible participants. This could not be avoided as it was only possible to identify eligible participants via this method and is mediated by the diverse sample of participants that we recruited into the study.

### Implications for health decision-makers

Overall, the additional eMCoC funding has been well received by both staff and service users. The implementation of the enhanced roles is perceived to have supported delivery of team-based care, facilitating successful release of midwifery capacity and the delivery of additional public health activities. Supporting a team-focused ethos seems an important feature of the enhanced service. This is consistent across sites and from both staff and service user perspectives.

Flexibility in the eMCoC specification guidance which permitted use of funds for a range of staff roles facilitated multiple eMCoC service types. This was a positive result with each service seeming to meet the essential functions and objectives of eMCoC as well as perceived local needs. The services were thus widely acceptable to staff and service users. This flexibility should be maintained and prioritised.

The flexible approach taken by enhanced teams to target service user groups should be supported. Sites clearly valued the focus on deprived population groups, but have equally strived to broaden the characteristics of focus to include a wider and more diverse set of social risk factors and vulnerabilities. In line with existing equity frameworks, a more inclusive and intersectional approach to targeting high-need populations could be championed in the way funding is allocated.

Implementing eMCoC was not without challenges. Many of the 58 teams initially funded were unable to implement eMCoC during the evaluation period because of institutional and organisational barriers. These barriers to implementation echo the wider barriers of implementing midwifery continuity of carer, described in a recent review.^29^

Attention should be placed on the barriers to implementation and sustainability issues which can be addressed, namely: delays in releasing funding from LMNS/Integrated Care Boards to providers and protecting MSW and midwifery time in their respective teams.

The eMCoC services have been built organically from ‘the ground up’. This should be learnt from considering what has worked well previously at a local level with attention paid to enablers of eMCoC services (i.e. characteristics of the enhanced role, skills set etc.). This should be considered in line with the above recommendation around flexibility. Relatedly, our conclusions point to the value of autonomy afforded to local areas to use enhancing funding to best suit the needs of their staff and specific service user groups, based on local needs. This trust and autonomy should be maintained going forward.

Due to the timeframes of this rapid, formative evaluation, longer-term clinical outcomes fell outside of the evaluation scope. Instead, we focused on developing a logic model for eMCoC, to identify process outcomes and potential key clinical outcomes which should be explored in a longer-term evaluation (see figure 1). Exploration of the evolution of local delivery as well as longer term sustainability should also be considered.

## Supplementary material

10.1136/bmjopen-2024-095509 online supplemental file 1

## Data Availability

No data are available.
