# Supplementary material for: Releasing time to deliver care: a mixed methods evaluation of the implementation of enhanced midwifery continuity of carer
Source: BMJ Open. 2025 Jul 28;15(7):e095509. doi: 10.1136/bmjopen-2024-095509 (PMC12306479; doi:10.1136/bmjopen-2024-095509)
Supplement: online supplemental file 1 [file bmjopen-15-7-s001.docx]

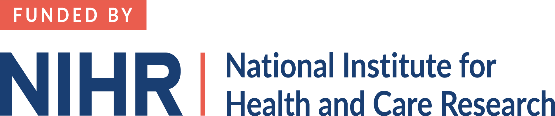

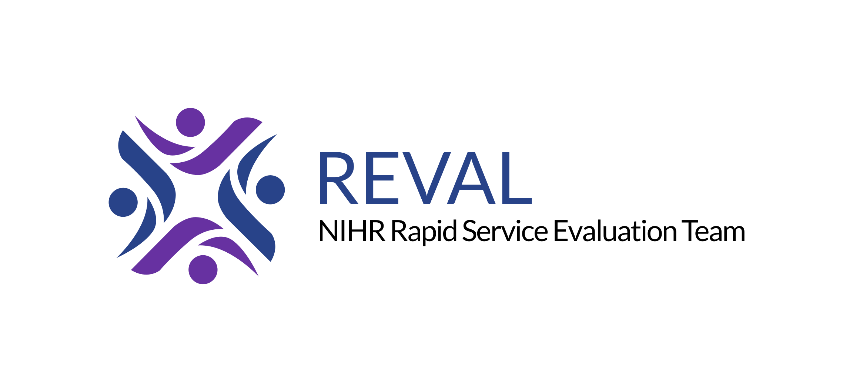


**Evaluation of the enhanced model of midwifery continuity of carer**

**Evaluation protocol summary | March 2023**

| **Title** | **Rapid evaluation of the enhanced model of midwifery continuity of carer** |
| --- | --- |
| **Background** | Midwifery Continuity of Carer (MCoC) aims to provide personalised and safe care to women and their families via provision of the same midwife, supported by a small team of midwives, throughout pregnancy, birth and the post-partum period.  NHS England is funding an enhanced MCoC model of care that aims to provide extra support to women and their families in the most deprived areas of England. Funding for enhanced MCoC has been allocated to 58 midwifery teams in 2022/23 with continued funding in 2023/24 (financial year). Funding is to be used, by these teams, to provide “holistic support that reduces midwives’ workload and releases additional time for the midwives to care for women” (NHS 2021). |
| **Aims** | Our overall aim is to undertake a rapid formative evaluation of enhanced MCoC implementation. We will generate rapid insights into the format of care delivery and the experiences of those delivering and receiving enhanced MCoC to assess its early impacts.  *Research questions*   1. Where are teams implementing the enhanced MCoC and are they focused on families in the highest decile of deprivation? 2. What are enhanced MCoC service delivery models and how have these been developed in response to service model guidance and related policies, existing MCoC services, high priority issues and specific local needs? 3. What are the barriers and facilitators to the implementation of enhanced MCoC models of care from a staff perspective and can fidelity to the model envisaged be maintained? 4. What are staff views on the acceptability of the enhanced elements of MCoC models and their experience of these, including how staff interface with other health and social care teams? 5. What evidence is there that delivery of enhanced MCoC is resulting in purposeful improvement to care delivery or leading to unintended consequences, including any early benefits or risk as judged by staff. 6. What factors are perceived to be linked to impacts of enhanced MCoC? 7. What are service user views on the acceptability of the enhanced elements of MCoC models and their experience of these? 8. What are the key theories of change, and themes within this, that are shaping current enhanced maternity continuity of carer service delivery models? 9. What may form the scope and design of a summative, longitudinal evaluation? |
| **Design** | Multi-site, multiple methodologies study. Interviews with staff, stakeholders and service users who are delivering or in receipt of the enhanced MCoC model, across nine case study sites. |
| **Sample** | The case site selection process may iterate based on information collected during the early stages of the evaluation, but we anticipate employing a maximum variation design to ensure meaningful variation in service types. Currently, based on scoping work, we anticipate case study sites being sampled to provide variation, where possible, in: geography; service model; previous experience in implementation of enhanced maternity services for disadvantaged and underserved groups; and local demography. |
| **Timelines** | Mapping of enhanced models and identification of case sites March 2023 to May 2023  In-depth exploration of the implementation and delivery of the enhanced model May to December 2023 (Workstreams 2 & 3) |
| **Funding** | This research is an independent evaluation undertaken by the NIHR Rapid Service Evaluation Team (REVAL). REVAL is funded via a competitive review process by the NIHR Health Services and Care Delivery Research Programme (NIHR151666). The views expressed in this protocol are those of the author(s) and not necessarily those of the NIHR, NHS England or the Department of Health and Social Care. |

# 
