# Supplementary material for: Releasing time to deliver care: a mixed methods evaluation of the implementation of enhanced midwifery continuity of carer
Source: BMJ Open. 2025 Jul 28;15(7):e095509. doi: 10.1136/bmjopen-2024-095509 (PMC12306479; doi:10.1136/bmjopen-2024-095509)
Supplement: Supplementary file 2 [file bmjopen-2024-095509.reviewer_comments.pdf]

PEER REVIEW HISTORY

BMJ Open publishes all reviews undertaken for accepted manuscripts. Reviewers are asked to complete a checklist review form and are provided with free text boxes to elaborate on their assessment. These free text comments are reproduced below.

ARTICLE DETAILS

Title (Provisional)

Releasing time to deliver care: A mixed methods evaluation of the implementation of enhanced midwifery continuity of carer

Authors

Gillibrand, Stephanie; Parkyn, Kate; Hall, Charlotte; Kletter, Maartje; Harkness , Elaine; Munford, Luke Aaron; Wilson, Paul; Dumville, Jo C.

VERSION 1 - REVIEW

**Reviewer**	**1**
**Name**	**McCourt, Christine**
**Affiliation**	**Department of Midwifery and Child health, City University London**
**Date**	**19-Feb-2025**
**COI**	**None**

This is an interesting study and was impressive in the level of detail covered in the article as well as the conduct and level of data collection. It covers an important and very current topic so should be seen as important for publication.

More detailed comments:

Introduction and background are reasonable given word limits that would prevent a more extensive account.

Methods looked clear and comprehensive – and an impressive amount of data collection was achieved, as well as challenges described.

Under staff it looks as though obstetricians or other medical staff were not interviewed and I wondered whether it was planned to include obstetricians, but they were not available? (This is something my team have experienced in other studies focused on midwifery care.)

Duration of interviews with women was quite short on average and I imagine there might have been quite a bit of variation, with some women more forthcoming than others – it might be good to add the range?

Analysis – the level of engagement with participants in this process was a positive feature. I wasn't clear what framework was being referred to (line 35-36) – was this using your initial logic model? CFIR?

The ways in which mixed methods were used – each analysis to inform the other – was also a positive feature.

The presentation of findings was clear and interesting. There is a lot packed in to a single article, covering experiences of the care from different perspectives as well as implementation challenges and solutions, so I wonder whether the research team have considered drafting a specific paper on implementation, to provide more detailed learning than could be covered here.

Principal findings

I was a little confused by this header initially but then realised it is an overview drawing together the main findings from each component of the work. Perhaps a sentence could be added at the beginning of the section to explain this, although it does become clear once reading the section.

On lines 40-41 it states this is the first study but I'm not sure that is correct as there was an evaluation of the LEAP programme – so perhaps first peer reviewed study? Or adjust wording to acknowledge the LEAP report. This would be worth citing in any case as it has very comparable themes and findings.

Typos

- Line 20 – rollout should be roll out

-

Reviewer 2

Name Tavella, Nicola

Affiliation Mount Sinai Hospital, Obstetrics, Gynecology, and Reproductive Science

Date 27-Mar-2025

COI None

This manuscript details the results of a mixed methods analysis of implementing an enhanced midwifery continuity of care (eMCoC) program across 58 clinical obstetric teams in the UK. The authors question whether such an intervention can be feasibly implemented across institutions such that care provided to pregnant patients is improved. I commend the

authors on a well-designed study examining a fundamental topic via innovative research methods. I recommend this article for minor revision for several reasons.

First, I found the introduction, while thought provoking, discusses "deprivation" too abstractly. Only in the last two paragraphs does the reader learn even how deprivation is being operationalized (via the Index of Multiple Deprivation). The introduction would be improved by discussing more concretely how deprivation is measured in this context, and how it relates to the process by which an eMCoC intervention could improve perinatal health.

Second, the statistical analysis, while appropriate, seemed incomplete. Specifically, for the quantitative analysis, it is mentioned that continuous variables will be displayed as means (sd) or medians (IQR). However, there was no reported test of normality to inform which variables should be displayed as which. I would recommend a Shapiro-Wilks test of normality to inform the presentation of all quantitative data.

Third, I feel that the authors do not adequately address the strengths and limitations of the study in the submitted manuscript. There are several sentences discussing the potential for sampling error in how cases were selected, which does not seem necessary if they take the place of discussing other limitations (selection bias in choosing cases, for example).

Finally, there are spelling and grammatical mistakes (including in the title) which should be addressed in a revised manuscript.

**Reviewer**	**3**
**Name**	**Schweitzer, Jane**
**Affiliation**	**Gold Coast Hospital and Health Service, Nursing Midwifery Education**
**Date**	**31-Mar-2025**
**COI**	**None**

Dear Authors,

Thank you for your commitment to publishing this valuable work.

Your commitment to bringing the findings to the attention of both teams at the coalface and policymakers is to be applauded.

I have enjoyed reading your work and am recommending some minor revisions to the potential impact of your paper.

I found your Logic model a particularly useful, this model captures the impacts of the eMCoC model in a way that is quite practical, and I believe could be used as helpful tool for those wanting to explore implementing an eMCoC service, as such I would recommend including it

in the body of the paper (practically speaking jumping between a supplemental file & the main paper may be onerous to some readers)

Finally, in your conclusion section there is an excellent description of next steps for policy makers & I wonder if a next steps for the local level (i.e. midwives & enhanced role holders) would also be beneficial (perhaps as a supll

****** The reviewer provided a marked copy with additional comments. Please contact the publisher for full details.******

VERSION 1 - AUTHOR RESPONSE

Reviewer: 1
Dr. Christine McCourt, Department of Midwifery and Child health
Comments to the Author:
This is an interesting study and was impressive in the level of detail covered in the article as well as the conduct and level of data collection. It covers an important and very current topic so should be seen as important for publication.

More detailed comments:

Introduction and background are reasonable given word limits that would prevent a more extensive account.

Methods looked clear and comprehensive – and an impressive amount of data collection was achieved, as well as challenges described.
Under staff it looks as though obstetricians or other medical staff were not interviewed and I wondered whether it was planned to include obstetricians, but they were not available? (This is something my team have experienced in other studies focused on midwifery care.)

We chose not to interview obstetricians as it wasn't clear that they played a substantive role in the focus of the care provided by enhanced teams specifically. Rather, as the focus on enhanced teams is relieving midwifery capacity and providing additional access to services via maternity support workers, we prioritised sampling of these roles, and within this, made efforts to sample different Banding of staff (e.g. HOM, DOMS, junior and more experienced midwives etc).

Duration of interviews with women was quite short on average and I imagine there might have been quite a bit of variation, with some women more forthcoming than others – it might be good to add the range?

Range of interviews has been added to page 8. We did find that some participants from some age cohorts were more forthcoming and therefore tended to be longer, and those with younger participants tended to be shorter. This is not unusual for qualitative data collection with these participant groups.

Analysis – the level of engagement with participants in this process was a positive feature. I

wasn't clear what framework was being referred to (line 35-36) – was this using your initial logic model? CFIR?

Here we are referring to an adapted thematic framework for coding and analysing the data. We have clarified this in the text.

The ways in which mixed methods were used – each analysis to inform the other – was also a positive feature.

The presentation of findings was clear and interesting. There is a lot packed in to a single article, covering experiences of the care from different perspectives as well as implementation challenges and solutions, so I wonder whether the research team have considered drafting a specific paper on implementation, to provide more detailed learning than could be covered here.

As mentioned in the paper, the characteristics of implementation, along with the challenges echo similarly those of implementing midwifery continuity of carer, especially as the pre-cursor to enhanced midwifery of continuity of carer is the delivery of continuity. As such, many of these characteristics and the challenges are already identified within existing literature, especially in recent reports on the implementation of MCoC. Therefore, we think it is better captured in this paper within the wider context and findings around the implementation of eMCoC.

Principal findings
I was a little confused by this header initially but then realised it is an overview drawing together the main findings from each component of the work. Perhaps a sentence could be added at the beginning of the section to explain this, although it does become clear once reading the section.

We've changed the heading to 'discussion' to make this clearer.

On lines 40-41 it states this is the first study but I'm not sure that is correct as there was an evaluation of the LEAP programme – so perhaps first peer reviewed study? Or adjust wording to acknowledge the LEAP report. This would be worth citing in any case as it has very comparable themes and findings.

We have added references to the LEAP study and indicated the differences between the programmes (i.e LEAP was targeted MCoC and the enhanced element not connected to the midwifery teams).

Typos
- Line 20 – rollout should be roll out

corrected

Reviewer: 2
Dr. Nicola Tavella, Mount Sinai Hospital
Comments to the Author:
This manuscript details the results of a mixed methods analysis of implementing an enhanced midwifery continuity of care (eMCoC) program across 58 clinical obstetric teams in the UK. The

authors question whether such an intervention can be feasibly implemented across institutions such that care provided to pregnant patients is improved. I commend the authors on a well-designed study examining a fundamental topic via innovative research methods. I recommend this article for minor revision for several reasons.
First, I found the introduction, while thought provoking, discusses "deprivation" too abstractly. Only in the last two paragraphs does the reader learn even how deprivation is being operationalized (via the Index of Multiple Deprivation). The introduction would be improved by discussing more concretely how deprivation is measured in this context, and how it relates to the process by which an eMCoC intervention could improve perinatal health.

We have provided some greater detail around the description of deprivation on page 6, including how deprivation is associated with perinatal mental health.

The IMD is a neighbourhood measure of relative deprivation for areas inhabited by approximately 1-3,000 people. The index is made up of 39 indicators spanning income, employment, education, skills and training, health, crime, barriers to housing and services, and the living environment. Deprivation is a known factor associated with adverse perinatal health outcomes (10). Drivers of these inequalities associated with living in a more deprived area include higher body mass index, smoking at birth, premature delivery, lower birth weight, lower breastfeeding rates etc (11-13).

However, as stated on page 5, “there is no identified academic evidence base that clearly identifies the eMCoC programmes and its mechanisms that may impact on these outcomes” and therefore we have referred to potential mechanisms to perinatal inequalities above.

Second, the statistical analysis, while appropriate, seemed incomplete. Specifically, for the quantitative analysis, it is mentioned that continuous variables will be displayed as means (sd) or medians (IQR). However, there was no reported test of normality to inform which variables should be displayed as which. I would recommend a Shapiro-Wilks test of normality to inform the presentation of all quantitative data.

We have presented the mean and the median for continuous variables. In addition, we have presented the standard deviation (of the mean) and the interquartile range (IQR) as measures of dispersion, We present both the mean and the median as they capture different aspects of quantitative continuous variables. For example, the mean can be susceptible to being influenced by outliers (e.g. values of the variable that are far away from others).

We are not suggesting that one measure (mean or median) is preferable to the other and thus have not tested this formally. Instead, we present both measures of the ‘average’ to give the reader the full information. We do not test for normality as we present the information descriptively – e.g. we do not perform and inferential analysis and hence the normality of the underlying distributions is not of primary interest.

The sample sizes are quite large in the descriptive analyses, and so the difference between the mean and median is relatively small as is common with a large N.

Third, I feel that the authors do not adequately address the strengths and limitations of the

study in the submitted manuscript. There are several sentences discussing the potential for sampling error in how cases were selected, which does not seem necessary if they take the place of discussing other limitations (selection bias in choosing cases, for example).

Thank you for the comment, we have aimed to clarify in the limitations section of the discussion about the number of sites that we were able to choose. And we have offered a reflection on the selection of individuals in those sites and how they were selected. We have amended the language to better reflect this.

Finally, there are spelling and grammatical mistakes (including in the title) which should be addressed in a revised manuscript.

We have undertaken another proof read and amended spelling and grammatical errors throughout.

Reviewer: 3
Dr. Jane Schweitzer, Gold Coast Hospital and Health Service
Comments to the Author:
Dear Authors,
Thank you for your commitment to publishing this valuable work.
Your commitment to bringing the findings to the attention of both teams at the coalface and policymakers is to be applauded.
I have enjoyed reading your work and am recommending some minor revisions to the potential impact of your paper.
I found your Logic model a particularly useful, this model captures the impacts of the eMCoC model in a way that is quite practical, and I believe could be used as helpful tool for those wanting to explore implementing an eMCoC service, as such I would recommend including it in the body of the paper (practically speaking jumping between a supplemental file & the main paper may be onerous to some readers)

The logic model has been added to the main file, page 19.

Finally, in your conclusion section there is an excellent description of next steps for policy makers & I wonder if a next steps for the local level (i.e. midwives & enhanced role holders) would also be beneficial (perhaps as a supll[note the end of this comment was cut off]

Since the paper was submitted, we have been informed that the funding of the service will be moved from central (national) allocation to local level commissioning. The current recommendations are aimed at this local level, which now has closer relevance to midwives and enhanced role holders through this shift in commissioning. We have amended the title to demonstrate the relevance of these recommendations to a wider audience than just policy-makers, i.e. health decision-makers, which is inclusive of local systems and teams.

The impact of this data "gets lost" using this large table only presentation; options could be to break the data into smaller bite size tables or use other visualisation of the information such as grouped by service delivery type.

We have broken up Table 5 to smaller tables (6 & 7) as requested in the comments on the manuscript. We have also cut the final table ‘pregnancy losses’ as upon revision we felt it was not relevant.

Consider inclusion of direct participant quotes in this section to bring additional power to the findings

We have now added participant quotes on pages 21 and service user quotes page 22

VERSION 2 - REVIEW

**Reviewer**	**1**
**Name**	**McCourt, Christine**
**Affiliation**	**Department of Midwifery and Child health, City University London**
**Date**	**31-May-2025**
**COI**	

Revisions have addressed the recommendations. Just on issue -

I didn't understand the use of 'Ecco’s' - this is an edit from eMCoC. What is Ecco?

**Reviewer**	**2**
**Name**	**Tavella, Nicola**
**Affiliation**	**Mount Sinai Hospital, Obstetrics, Gynecology, and Reproductive Science**
**Date**	**09-Jun-2025**
**COI**	

Thank you very much to the authors for thoughtfully revising this manuscript. I feel that the revisions sufficiently improve the study such that it is now suitable for publication in this journal, and anticipate it will contribute importantly to improving perinatal outcomes through the incorporation of midwifery care.

**Reviewer**	**3**
**Name**	**Schweitzer, Jane**

Affiliation **Gold Coast Hospital and Health Service, Nursing Midwifery Education**

Date **14-Jun-2025**

COI

Thank you for taking on board the suggestions of the reviewers. I thought the change to the tables worked well & the additional description for Figure 1 helpful. The quotes from interviewees added weight to the descriptive analysis.
